# Utilization of paediatric and general medical services by children and adolescents in Germany. Results of the cross-sectional KiGGS Wave 2 study and trends

**DOI:** 10.17886/RKI-GBE-2018-099

**Published:** 2018-12-12

**Authors:** Stefanie Seeling, Franziska Prütz, Johanna Gutsche

**Affiliations:** Robert Koch Institute, Berlin, Department of Epidemiology and Health Monitoring

**Keywords:** CHILDREN AND ADOLESCENTS, UTILIZATION OF OUTPATIENT SERVICES, PAEDIATRICS, GENERAL MEDICINE, HEALTH MONITORING

## Abstract

In Germany, specialists in paediatrics and general medicine in private practices provide the bulk of outpatient treatment. Data from the second wave of the German Health Interview and Examination Survey for Children and Adolescents (KiGGS Wave 2, 2014-2017) surveyed the ambulatory attendance of paediatric and general medical services for 0- to 17-year-old children and adolescents. During the last 12 months, 72.8% of girls and 72.7% of boys have made use of outpatient paediatric treatment and 25.9% of girls and 24.6% of boys have made use of outpatient general medical services. Attendance rates in paediatric practices decrease with age, whereas those of general medical practices increase. While no relevant differences between genders exist, rural areas evidence significantly lower paediatric practice and significantly higher general medical practice attendance rates. Compared to the data collected in the previous KiGGS studies, the trend over the last ten years approximately indicates an increase in the use of paediatric services and a decrease in the use of general medical services.

## Introduction

In Germany, basic outpatient treatment for children and adolescents is provided mainly by paediatricians in private practice. In particular in rural areas, specialists in general medicine also provide services for children and adolescents [1-3]. The prevention of diseases and development-related disorders is prioritised in the provision of care given to children and adolescents. Of great importance here are the early detection examinations (called U-Untersuchungen in Germany) for children and adolescents [4, 5] regulated by the Federal Joint Committee (G-BA), as well as the vaccinations recommended [6] by the Standing Committee on Vaccination (STIKO) at the Robert Koch Institute. Surveys indicate that in the age groups of infants and small children, who are in the focus of most interventions, paediatricians attend nearly all children over a one year period. A further trend is that with age the proportion of children and adolescents that are attended in paediatric practices gradually decreases, in favour of general medicine practices [1, 2].

Data from the second wave of the German Health Interview and Examination Survey for Children and Adolescents (KiGGS Wave 2, 2014-2017) allows up-to-date analyses on the utilization of paediatric and general medical services. In addition to the assessments of prevalences (frequencies), number of contacts and possible influencing factors such as gender, place of residence and socioeconomic status, this article also compares the results with those from previous KiGGS survey points (trends). Figures indicating the utilization of early detection examinations by children are reported in a further article of this issue of the Journal of Health Monitoring.

**KiGGS Wave 2**

Second follow-up to the German Health Interview and Examination Survey for Children and Adolescents

**Data owner:** Robert Koch Institute

**Aim:** Providing reliable information on health status, health-related behaviour, living conditions, protective and risk factors, and health care among children, adolescents and young adults living in Germany, with the possibility of trend and longitudinal analyses

**Study design**: Combined cross-sectional and cohort study

**Cross-sectional study in KiGGS Wave 2**

**Age range:** 0-17 years

**Population:** Children and adolescents with permanent residence in Germany

**Sampling:** Samples from official residency registries - randomly selected children and adolescents from the 167 cities and municipalities covered by the KiGGS baseline study

**Sample size:** 15,023 participants

**KiGGS cohort study in KiGGS Wave 2**

**Age range:** 10-31 years

**Sampling:** Re-invitation of everyone who took part in the KiGGS baseline study and who was willing to participate in a follow-up

**Sample size:** 10,853 participants

**KiGGS survey waves**

► KiGGS baseline study (2003-2006), examination and interview survey
► KiGGS Wave 1 (2009-2012), interview survey
► KiGGS Wave 2 (2014-2017), examination and interview survey

More information is available at www.kiggs-studie.de/english

## Indicator

KiGGS is part of the health monitoring system at the Robert Koch Institute and includes repeated cross-sectional surveys of children and adolescents aged 0 to 17 (KiGGS cross-sectional study) that are representative for Germany. The KiGGS baseline study (2003-2006) was conducted as an examination and interview survey, the first follow-up study (KiGGS Wave 1, 2009-2012) as a telephone-based interview survey and KiGGS Wave 2 (2014-2017) as an examination and interview survey.

A detailed description of the methodology is contained in New data for action. Data collection for KiGGS Wave 2 has been completed in issue S3/2017 as well as KiGGS Wave 2 cross-sectional study – participant acquisition, response rates and representativeness in issue 1/2018 of the Journal of Health Monitoring [7, 8].

The utilization of outpatient paediatric and general medicine services was surveyed in KiGGS Wave 2 through a questionnaire filled out in writing. Legal guardians of 0- to 13-year-old children were asked: ‘Please tell us which doctors in private practice of the following disciplines you have consulted for your child in the last 12 months and how often’. Contacts in the context of early detection examinations were also to be reported; home visits by doctors were explicitly included (‘Please include home visits’). The possible answers included: ‘paediatrician’ and ‘general practitioner’ (editorial note: in the German version of the questionnaire, the latter category was divided into ‘general practitioner’ and ‘specialist in general medicine’ according to the relevant physician groups in general medicine with and without specialisation in their area of practice). 14- to 17-year-old adolescents answered a correspondingly adapted question by themselves.

The analyses are based on data from 14,468 adolescents (7,298 girls, 7,170 boys) aged 0 to 17 with valid data on the use of outpatient medical services. The results are presented as prevalences and are stratified by gender, age and size of municipality, further analyses are provided for socio economic status (SES) [9].

The calculations were carried out using a weighting factor that corrects deviations within the sample from the population structure with regard to regional structure (rural area/urban area), age (in years), gender, federal state (as at 31 December 2015), German citizenship (as at 31 December 2014) and the parents’ level of education (Microcensus 2013 [10]). Trends across the three KiGGS waves were described using age-standardised prevalences for the three data collection points that were calculated based on the population of 31 December 2015. Univariate logistic regression was applied to analyse a linear trend across survey waves.

This article considers prevalences for the utilization of outpatient medical services during the last 12 months with 95% confidence intervals (95% CI). For children and adolescents who have availed themselves of services, the arithmetic mean for the amount of approaches with paediatricians and general medicine doctors is provided. Prevalences are estimates, the precision of which can be assessed through the use of confidence intervals; wide confidence intervals thereby indicate a greater statistical uncertainty in the results. A statistically significant difference between groups is assumed when the corresponding p-value is smaller than 0.05. For reasons of improved readability, this article discusses specialists in general medicine without explicitly referring to the far smaller group of general practitioners, who are, however, included in the analyses.

## Results and discussion

According to the results from KiGGS Wave 2, 72.8% of girls and 72.7% of boys have received outpatient paediatric services over the last 12 months (Table 1). In the group of 0- to 2-year-olds, nearly all children (girls 97.1% and boys 96.9%) were attended by a paediatrician. This proportion decreases with age and for 14- to 17-year-old adolescents drops to 39.7% of girls and 38.9% of boys.

While there are no differences regarding gender in the utilization of paediatric services, there are clear differences with regard to the place of residence: children and adolescents from rural areas and small towns with less than 20,000 inhabitants use paediatric services less frequently than their urban peers (Table 1). Furthermore, girls and boys with low and medium SES visit a paediatrician statistically significantly less often than those with high SES.

**Figure 1:**
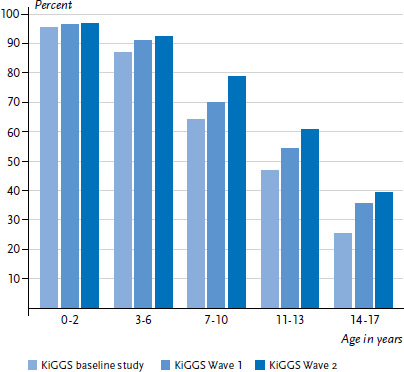
12-month prevalence of outpatient utilization of paediatric services according to age in comparison to previous KiGGS waves (KiGGS baseline study n=8,504 girls, n=8,832 boys; KiGGS Wave 1 n=5,972 girls, n=6,130 boys; KiGGS Wave 2 n=7,298 girls, n=7,170 boys) Source: KiGGS baseline study (2003-2006), KiGGS Wave 1 (2009-2012), KiGGS Wave 2 (2014-2017)

The proportion of children and adolescents who used paediatric services during the last 12 months has increased significantly over the course of the last ten years (Figure 1). While in the KiGGS baseline study (2003-2006) the figures were 61.4% of girls and 62.1% of boys, this proportion rose to 67.9% of girls and 69.2% of boys in KiGGS Wave 1 (2009-2012). The additional increase to 72.8% of girls and 72.7% of boys in KiGGS Wave 2 reveals a significant linear trend across the three survey points. Stratified by age groups, this trend is visible for children over three and adolescents, whereas there is no significant development over time for 0- to 2-year-old children.

The average number of contacts made to paediatricians during the last 12 months was 3.8 for girls and 3.7 for boys in KiGGS Wave 2 (Table 2). The mean contact frequency was highest for the 0- to 2-year-old age group and lowest for the 11- to 13-year-old age group. Compared to previous KiGGS survey points no significant trend of either an increase or decrease of the contact frequency is evident.

Children and adolescents also avail themselves of general medical services. KiGGS Wave 2 reveals that during the last 12 months 25.9% of girls and 24.6% of boys (Table 1) made use of such services during the last 12 months. This proportion increases with age from 8.5% of girls and 11.9% of boys in the 0- to 2-year age group to 47.4% of girls and 44.7% of boys in the 14- to 17-year age group.

There is no significant difference in the utilization of general medical services between genders (Table 1). Place of residence, however, does play a clear role: specialists in general medicine attend children and adolescents particularly frequently in rural areas. SES too plays a role. Girls with low and medium SES are attended by specialists in general medicine more frequently compared to those with high SES (26.2% vs. 27.9% vs. 19.4%). The figures for boys with low, medium and high SES are 23.3%, 26.7% and 18.8% respectively, however, only the differences between medium and high SES are significant. The focus article on social differences in the utilization of medical services in this issue presents the results of a multivariate analysis. When age and migration background are controlled, the difference between the low and the high status group becomes statistically significant for boys too [11].

**Figure 2:**
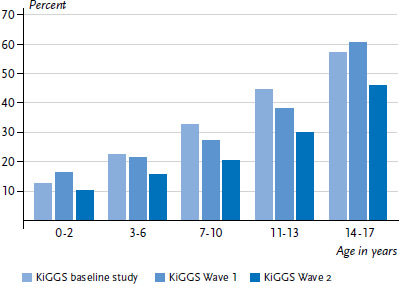
12-month prevalence of outpatient utilization of general medical services (specialists in general medicine and general practitioners) according to age in comparison to previous KiGGS waves (KiGGS baseline study n=8,505 girls, n=8,832 boys; KiGGS Wave 1 n=5,955 girls, n=6,102 boys; KiGGS Wave 2 n=7,298 girls, n=7,170 boys) Source: KiGGS baseline study (2003-2006), KiGGS Wave 1 (2009-2012), KiGGS Wave 2 (2014-2017)

Over the course of the last ten years, the proportion of children and adolescents who have availed themselves of general medical services during the last 12 months has decreased significantly (Figure 2). In the KiGGS baseline study this proportion was 35.4% (girls 35.5% and boys 35.3%), in KiGGS Wave 1 33.8% (girls 34.0% and boys 33.6%) and in KiGGS Wave 2 clearly lower at 25.2% (girls 25.9% and boys 24.6%). Differentiating the development over time by age, this trend is significant for girls and boys of the age groups 3 to 6 years and older.

According to KiGGS Wave 2 data, the average number of contacts made to specialists in general medicine during the last 12 months was 3.0 for girls and 2.6 for boys (Table 2). The mean contact frequency is highest for 14- to 17-year-old girls. For boys, the highest contact frequency is found in the 0- to 2-year and 14- to 17-year age groups. Across the KiGGS survey points, no significant linear trend for the contact frequency is evident in either gender.

In total, 12.1% of children and adolescents did not use any outpatient paediatric or general medical services during the last year. This proportion rises with age from around four percent of 3- to 6-year-old girls and boys to around one quarter of 14- to 17-year-olds. As regards the medical care needs of the particularly relevant age group of 0- to 2-year-olds, the number of cases in the subgroup that received no medical services from either paediatricians or general medicine specialists is too small to give reliable prevalence estimates.

KiGGS survey results show the high coverage rates for infants and small children aged under three, as well as an increase in the rates of attendance by paediatricians for children over three and adolescents over the last ten years. The average number of contacts did not increase over this period.

The reasons for the increase in the utilization of paediatric services are undoubtedly complex. One factor is presumably the introduction of new early detection examinations (U7a, U10, U11, J2) in 2006 and increased participation rates due to the introduction of new invitation, reminder and feedback systems in Germany’s federal states [12, 13]. Moreover, since the KiGGS baseline study, the STIKO recommendations have included additional vaccinations such as against human papillomavirus (HPV) that go along with corresponding consultation needs [14, 15]. The spectrum of diseases has shifted towards chronic diseases and developmental and behavioural disorders [16, 17], which has been described as ‘new morbidity’ and is accompanied by an increased need for consulting and treatment [18]. In general too, paediatricians have highlighted the increased parental need for information [18].

Attendance of children and adolescents by specialists in general medicine shows a contrasting development to attendance by paediatricians. During the last ten years, attendance of children and adolescents by general medicine specialists has seen a clear decline. The increase observed in KiGGS Wave 1 for the 14- to 17-year age group [2] did not continue and has instead reversed into a marked decrease. KiGGS Wave 2 data confirms the findings of previous KiGGS waves that the role of general medicine in the provision of medical care to children and adolescents increases with age and that specialists in general medicine play a more important role in rural than urban areas [1, 2]. Analyses of the statutory health insurance company Barmer GEK confirm this finding. Accordingly, besides age, a key factor in selecting a doctor is regional availability [3].

KiGGS Wave 2 data indicates no significant differences between genders in the rate of utilization of paediatric or general medical services. For girls at adolescence, however, the contact frequency to doctors overall and the number of physician groups contacted increases compared to boys [1, 2], with gynaecological issues and contraception playing an important role [19, 20].

By SES, the results indicate a higher use of paediatric and a lower use of general medical services by children and adolescents with high SES. As analyses so far do not consistently confirm this finding as a separate influencing factor, further analyses of correlations will be needed which also factor in the place of residence [2, 21].

The present analyses of the utilization of paediatric and general medical services from the perspective of parents of children aged up to 13 and of adolescents themselves provide important information that goes beyond that found in official statistics and claims data. Robert Koch Institute health surveys contribute data for the statutory and privately health insured and allows links with social and other influencing factors to be created. The descriptive analyses on the utilization of services, however, cannot contribute to the discussion about the appropriateness of health care for children and adolescents in general medical practices [22, 23], particularly because the survey did not collect data regarding the reason for seeking treatment. A European level project promises to provide important insights in this regard. The Models of Child Health Appraised (MOCHA) project analyses the advantages and disadvantages of different models of primary care for children in Europe: primarily through paediatricians, through general practitioners or mixed paediatric and general practitioner care. First results are expected for 2018 [24, 25].

How frequently children and adolescents contact both doctor groups, paediatricians and general medicine specialists, or do not see a doctor at all, and which factors are related to the decision as to which doctor to consult will be a question for further analyses. Deeper level analyses will need to consider factors such as overall health and health behaviour, but also the regional spread of doctors and availability of medical practices [26], plus further sociodemographic factors such as migration background. The Behavioral Model of Health Services Use according to Andersen, which divides the factors that influence the utilization of health services into the categories of Predisposing Characteristics, Enabling Resources and Needs, could offer a basic framework [27]. Data from the KiGGS cohort also allows for longitudinal analyses that make it possible to draw conclusions on the utilization of services over the life course.

## Key statements

- Over the course of one year, almost three quarters of all children and adolescents visit a paediatric and around one quarter a general medical practice.
- Paediatric services are attended less frequently with age while attendance rates in general medical practices rise.
- In rural areas, general medical services are used more frequently and paediatric services less frequently than in cities.
- Since the KiGGS baseline study (2003-2006), the utilization of paediatric services has increased and the utilization of general medical services has decreased.

## Figures and Tables

**Table 1:** 12-month prevalence of outpatient utilization of paediatric and general medical services (specialists in general medicine and general practitioners) by children and adolescents according to age, size of municipality and socioeconomic status (n=7,298 girls, n=7,170 boys) Source: KiGGS Wave 2 (2014-2017)

Paediatricians					Specialists in general medicine, General practitioners			
Girls			Boys		Girls		Boys	
%		(95 % CI)	%	(95 % CI)	%	(95 % CI)	%	(95 % CI)
**Total**	**72.8**	**(70.8-74.7)**	**72.7**	**(70.9-74.4)**	**25.9**	**(23.5-28.5)**	**24.6**	**(22.2-27.1)**
**Age group**								
0-2 Years	97.1	(94.3-98.6)	96.9	(94.3-98.4)	8.5	(6.0-11.9)	11.9	(8.6-16.1)
3-6 Years	93.4	(91.2-95.2)	91.7	(89.3-93.6)	16.5	(13.5-19.9)	14.9	(12.2-18.1)
7-10 Years	79.1	(76.2-81.7)	78.8	(75.8-81.5)	20.9	(17.8-24.4)	20.1	(16.9-23.8)
11-13 Years	59.9	(56.0-63.7)	61.7	(57.5-65.7)	31.8	(27.8-36.1)	28.4	(24.8-32.3)
14-17 Years	39.7	(36.1-43.5)	38.9	(35.2-42.7)	47.4	(44.0-51.0)	44.7	(41.3-48.2)
**Size of municipality**								
Rural (<5,000 inhabitants)	65.3	(60.3-69.9)	65.7	(60.6-70.5)	44.9	(37.9-52.1)	38.3	(32.2-44.8)
Small town (5,000-<20,000 inhabitants)	68.8	(64.9-72.5)	68.9	(65.6-71.9)	30.0	(26.6-33.7)	31.0	(26.7-35.7)
Middle-sized town (20,000–<100,000 inhabitants)	74.9	(71.9-77.8)	74.5	(72.0-76.9)	19.6	(16.7-22.8)	19.2	(16.2-22.6)
Large cities (≥100,000 inhabitants)	78.8	(75.9-81.4)	78.4	(75.4-81.1)	17.4	(14.9-20.2)	16.3	(13.6-19.5)
**Socioeconomic status**								
Low	70.3	(65.7-74.6)	70.4	(65.8-74.7)	26.2	(22.6-30.2)	23.3	(19.8-27.3)
Medium	72.1	(69.8-74.3)	72.0	(69.6-74.2)	27.9	(25.0-31.0)	26.7	(23.9-29.8)
High	77.4	(74.5-80.0)	77.9	(75.5-80.1)	19.4	(16.4-22.8)	18.8	(16.0-21.9)

CI=Confidence interval

**Table 2:** Number of contacts (arithmetic mean) made to paediatricians and specialists in general medicine (including general practitioners) during the last 12 months according to gender and age Source: KiGGS Wave 2 (2014-2017)

Paediatricians				Specialists in general medicine, General practitioners		
n		Number of contacts	(95% CI)	n	Number of contacts	(95% CI)
**Girls (total)**	**5,020**	**3.8**	**(3.6-4.0)**	**1,872**	**3.0**	**(2.8-3.2)**
**Age group**						
0-2 Years		6.2	(5.6-6.8)		2.5	(2.0-3.1)
3-6 Years		3.7	(3.5-4.0)		2.7	(2.3-3.0)
7-10 Years		2.6	(2.4-2.7)		2.2	(2.0-2.5)
11-13 Years		2.5	(2.3-2.7)		2.5	(2.2-2.8)
14-17 Years		3.3	(2.9-3.7)		3.7	(3.4-4.0)
						
**Boys (total)**	**4,999**	**3.7**	**(3.6-3.8)**	**1,714**	**2.6**	**(2.5-2.8)**
**Age group**						
0-2 Years		6.1	(5.7-6.4)		3.0	(2.2-3.7)
3-6 Years		3.8	(3.6-4.0)		2.5	(2.2-2.9)
7-10 Years		2.7	(2.5-2.8)		2.3	(2.0-2.5)
11-13 Years		2.5	(2.3-2.7)		2.1	(2.0-2.3)
14-17 Years		2.6	(2.3-2.9)		3.0	(2.7-3.3)
**Total (girls and boys)**		**3.7**	**(3.6-3.9)**		**2.8**	**(2.7-2.9)**

CI=Confidence interval
